# Vision-Related Quality of Life and Treatment Satisfaction Following Panretinal Photocoagulation in Diabetic Retinopathy—A Panel Study

**DOI:** 10.3390/medicina58121741

**Published:** 2022-11-28

**Authors:** Jelena B. Vasilijević, Igor M. Kovačević, Zoran M. Bukumirić, Gorica D. Marić, Nikola A. Slijepčević, Tatjana D. Pekmezović

**Affiliations:** 1 Clinic for Eye Diseases, University Clinical Centre of Serbia, 11000 Belgrade, Serbia; 2 Faculty of Medicine, University of Belgrade, 11000 Belgrade, Serbia; 3 Institute for Medical Statistics and Informatics, Faculty of Medicine, University of Belgrade, 11000 Belgrade, Serbia; 4 Institute of Epidemiology, Faculty of Medicine, University of Belgrade, 11000 Belgrade, Serbia; 5 Clinic for Endocrine Surgery, University Clinical Centre of Serbia, 11000 Belgrade, Serbia

**Keywords:** panretinal photocoagulation, diabetic retinopathy, vision-related quality of life, treatment satisfaction, RetTSQ

## Abstract

*Background and Objectives*: The aim of the study was to evaluate vision-related quality of life (VR-QOL) and treatment satisfaction (TS) in patients with diabetic retinopathy treated with panretinal photocoagulation (PRP). *Material and Methods*: The panel study included 95 patients who underwent PRP for diabetic retinopathy. Eligible patients with no history of previous PRP were interviewer-administered the National Eye Institute Visual Function Questionnaire (NEI VFQ-25) and Retinopathy Treatment Satisfaction Questionnaire (RetTSQ) beforehandand one month after the last session of laser application. The study was conducted between June 2017 and June 2019 at tertiary care center in Serbia, Belgrade. We assessed pre- to post-PRP values of the composite score and subscale scores of VFQ-25 and RetTSQ, using a paired samples *t*-test. Univariate logistic regression was used to analyze the relationship between binary outcomes and potential predictors. Multivariate regression included predictors from univariate analyses that were statistically significant. *Results*: The mean VFQ-25 composite score was 65.4 ± 17.4 before and 63.3 ± 19.5 after PRP (*p* = 0.045). Subscale analysis showed that two of the 11 items achieved a significant decrease after laser application (general vision and dependency). The mean RetTSQ score at baseline was 60.0 ± 11.8 and at the exit visit was 60.3 ± 12.3 (*p* = 0.858). Sub-scale analysis showed significant deterioration for five of the 13 items. Multivariate logistic regression found that significant predictor of VFQ-25 composite score reduction was fewer laser burns (*p* = 0.002) while significant predictor of RetTSQ total score reduction was presence of hyperlipidaemia (*p* = 0.021). *Conclusion*: The use of vision-related quality of life and treatment satisfaction questionnaires in conjunction with clinical examination, appears to provide a more comprehensive overview of an individual’s daily well-being following PRP. Laser treatment for diabetic retinopathy leads to deterioration of some of the patients’ perceived VR-QOL and TS. Health-care providers should inform patients about their treatment options and together decide which therapeutic method is best for them.

## 1. Introduction

Diabetic retinopathy (DR) is the most common microvascular complication of diabetes, and represents the major cause of acquired vision loss and blindness in working age population [1,2]. In its early non-proliferative stages, there are mild visual symptoms. As the disease progresses to more advanced levels (proliferative DR, PDR), a significant loss of vision can occur [3]. Over the past years, it has been established that timely panretinal photocoagulation (PRP) can prevent vision loss in DR and has since become the gold standard for treatment of PDR [4,5]. Without treatment, almost 50% of patients with PDR experience severe vision loss within five years. However, this treatment may be accompanied by a broad spectrum of vision-related side effects including restriction in peripheral visual fields, decrease in contrast sensitivity, accommodative defects and mild vision changes [6,7,8,9].

Treatment satisfaction (TS) is a subjective assessment of individual’s emotional and physical experiences with the received treatment, including the procedure and its results [10,11]. Frequently, physicians and patients do not have a same view of the treatment success. Every patient weighs the significant aspects of their treatment and determines his or her overall extent of satisfaction [12], which does not necessarily correspond to the objective treatment goal and visual outcome. Patients’ satisfaction influences treatment-related behaviors that in turn, have a strong impact on treatment success [13]. On the other hand, vision-related quality of life (VR-QOL) is related to the visual function and represents a degree to which vision affects an individual’s ability to complete activities of daily living such as reading, using a computer and driving [14]. 

Given that the effectiveness of treatment of PDR on patient-centered outcomes is scarce, we undertook this study to quantitatively examine the effect of PRP on different aspects of VR-QOL and TS by means of the NEI VFQ-25 and RetTSQ).

## 2. Materials and Methods

### 2.1. Study Design and Population

The panel study was conducted at the Eye Clinic of the University Clinical Centre of Serbia in Belgrade, Serbia, from June 2017 until June 2019. We included in the study 95 consecutive patients with bilateral DR that were treated with PRP. All patients signed informed consent according to the institutional guidelines. This study was part of a doctoral dissertation which was approved by the Ethics Committee of the Faculty of Medicine, University of Belgrade, record number 1550/II-2.

The sample size required to detect the effect size of 0.4 with *t*-test for dependent samples, based on a power of 0.95 and significance level of 0.05 was determined to be 84 subjects. The calculation of the sample size was done using the program G*Power 3.1.9.7 ((Heinrich Heine University, Düsseldorf, Germany).

#### 2.1.1. Phase 1: Data Collection

The following data were collected: socio-demographic (including age, gender, education, marital status and occupation) and health information (diabetes type, duration of disease, therapeutic regimen, most recent glycated haemoglobin). Demographic information and data on diabetes and microvascular complications were collected from medical records and an interview with the participant. The inclusion criteria included patients with diabetes older than 18 years, Serbian speaking, with diagnosis of DR that required PRP according to the criteria of the Early Treatment of Diabetic Retinopathy study [5]. The exclusion criteria were clinically significant coexisting ocular pathology such as glaucoma and age-related macular degeneration and previous treatment for DR. All patients underwent a complete ophthalmologic examination. The classification of DR and diabetic maculae oedema (DMO) were set using the International Clinical Classification System and the Early Treatment Diabetic Retinopathy Study Research Group criteria, respectively [5,15]. All patients had their visual field examined with the standard Threshold C 24-2 program of the Humphrey field analyzer. The results were depicted as mean deviation (MD) and pattern standard deviation (PSD). Central macular thickness (CMT) was obtained using optical coherence tomography (Copernicus, Optopol, Inc., Depew, NY, USA) taken at pre-laser baseline, and one month after laser treatment (Appendix A).

#### 2.1.2. Phase 2: Procedures

Scatter PRP was performed with a 532 nm Green Diode retinal laser (Ellex Laserex Integre 532 Green Diode Retinal Slit Lamp Laser). A multispot array with 200 μm spot size, 20 ms pulse duration, and 1.5-width spot spacing was used. On average, patients received between 1500–3000 burns of moderate intensity per eye, in three consecutive sessions, according to the ETDRS guidelines [5]. A retinal specialist delivered all laser sessions under topical anesthesia. Both eyes were treated simultaneously, which is the standard protocol for bilateral PDR. After the end of the first PRP session, the questionnaires were administered to the patients included in our study. One month after the last treatment, patients who completed PRP were administered the post-treatment questionnaires.

#### 2.1.3. Phase 3: Study Instruments

The National Eye Institute 25-Item Visual Function Questionnaire (NEI VFQ-25) is one of the most widely used tools in health service researches, along with the Retinopathy Treatment Satisfaction Questionnaire (RetTSQ) that had been already translated and validated into Serbian language at the time of our study [16,17]. It consists of 25 vision-targeted questions plus an additional general health-rating question. The questions fall into 11 vision-dependent subscales: general vision, ocular pain, near activities, distance activities, vision-specific social functioning, vision-specific mental health, vision-specific role difficulties, vision-specific dependency, driving, color vision and peripheral vision. Each item is coded in a scoring system ranging from 0 to 100. A score of 0 indicates the worst possible score, while a score of 100 indicates the best possible score [18,19]. 

The Retinopathy Treatment Satisfaction Questionnaire is a tool that was designed to measure satisfaction with the treatment of DR [2]. The RetTSQ covers 13 items addressing specific aspects of treatment satisfaction. Items are scored on a seven-point scale from 6 (very satisfied) to 0 (very dissatisfied). Treatment satisfaction is assessed as the sum of the scores of the 13 questions, with the total score ranging from 0 to 78, where a higher score indicates a greater satisfaction with the treatment. The RetTSQ items ask the following about the treatment: item 1: Overall satisfaction; item 2: How well it works; item 3: Side effects; item 4: Discomfort/pain; item 5: Unpleasantness; item 6: Ease/difficulty; item 7: Apprehension; item 8: Patient influence; item 9: Safety; item 10: Time-taken; item 11: Received information; item 12: Encouraging others; and item 13: Continuity. Access to Questionnaire RetTSQ: visit https://www.healthpsychologyresearch.com/information/currently-available-translated-questionnaires/rettsqs-retinopathy-treatment-satisfaction, accessed on 24 November 2022 (Appendix B).

### 2.2. Statistical Analysis

Arithmetic mean with standard deviation and n (percent) were used for description depending on the type of variable. To evaluate the effect of PRP on vision-targeted VR-QOL and treatment satisfaction, we performed comparison between pre- to post-PRP values of composite score and subscale scores of VFQ-25 and RetTSQ, with the paired samples *t*-test. Univariate logistic regression was used to analyse the relationship between binary outcomes (decrease in total score NEI VFQ-25 and RetTSQ) and potential predictors (socio-demographic, health and clinical parameters). Multivariate regression models included predictors from univariate analyses that were statistically significant at a significance level of 0.2. All *p* values less than 0.05 were considered significant. Statistical data analysis was performed using IBM SPSS Statistics 22 (SPSS Inc., Chicago, IL, USA).

## 3. Results

### 3.1. Characteristics of the Participants

The study included 95 patients with bilateral DR with a mean age of the respondents of 57.4 ± 13.2 years. Among them, 70 (73.7%) were men, and 25 (26.3%) were women. The majority (84.2%) had type 2 diabetes, while the others had type 1 diabetes (15.8%). The mean duration of a history of diabetes was 15.76 ± 9.36 years and mean recent glycosylated haemoglobin (HbA1c) was 8.6 ± 1.7%. The most common associated conditions were hypertension and hyperlipidaemia (83.2% and 48.4%, respectively). The most prevalent microvascular complication was polyneuropathy (41.1%) while nephropathy was present in 31.6% of the sample. Other demographic and clinical data for the participants are summarized in Table 1. 

Regarding DR severity, 5.3% of patients had very severe non proliferative DR, while the rest had proliferative DR (94.7%); of which 20% had no high-risk criteria PDR, 45.3% had high-risk PDR and 29.5% had advanced PDR. Among the 95 patients, 55% had diabetic macular oedema. 

### 3.2. BCVA, CMT and Visual Field Changes

Pre-treatment best corrected visual acuity (BCVA), measured by means of the LogMAR units, for the better-seeing eye was 0.30 ± 0.34, while BCVA following pan scatter laser treatment showed a significant decrease 0.37 ± 0.34 (*p* < 0.001, paired *t*-test). On the other hand, when comparing the best corrected visual acuity of the worse-seeing eye before and after laser treatment, there was no statistically significant difference (*p* = 0.368). In addition, macular thickness and volume significantly increased in the PRP treated subjects compared to the pre-treatment group (289.37 µm versus 312.47 µm; *p* < 0.001). One month after laser PRP, MD reduced from −7.40 ± 4.29 dB to −8.43 ± 4.81 dB (*p* < 0.001) and PSD worsened from 4.73 ± 2.52 dB to 5.11 ± 2.22 dB (*p* = 0.013). 

### 3.3. Quality of Life and Treatment Satisfaction Assessment

The mean NEI VFQ-25 composite score for the whole study sample was 65.4 ± 17.4 before laser treatment, and 63.3 ± 19.5 after treatment (*p* = 0.045, paired *t*-test). Subscale analysis showed that two of the 11 vision-related items reached statistically significant deterioration before and after laser treatment (general vision, *p* < 0.001 and dependency, *p* = 0.037); while none of the other subscale scores showed any significant differences before and after intervention, as shown in Table 2.

The total mean RetTSQ score at baseline was 60.0 ± 11.8, while the total mean RetTSQ score at the exit visit, was 60.3 ± 12.3, which was not statistically significant (*p* = 0.858, paired *t*-test). Sub-scale analysis showed a statistically significant change (deterioration) for five of the 13 items of RetTSQ (overall satisfaction with current treatment, discomfort/pain, unpleasantness, patient influence on the treatment and received information about treatment), as shown in Table 3.

Logistic regression analysis was performed with the change (decrease in score) in relation to baseline values, as the dependent variable, for the composite NEI VFQ-25 and RetTSQ total score after PRP. The multivariate logistic regression model, with the existence of composite NEI VFQ-25 score reduction as the dependent variable, included all those predictors that were statistically significant at the significance level of 0.2 in univariate models. The whole model (with all predictors) was statistically significant (*p* < 0.001). There was no significant multicollinearity between the predictors. In the multivariate logistic regression model, statistically significant predictor of composite NEI VFQ-25 score reduction was fewer laser burns (B = −0.003; *p* = 0.002). The models predictors are listed in Table 4. 

The multivariate logistic regression model, with the existence of total RetTSQ score reduction as the dependent variable, included all those predictors that were statistically significant at the significance level of 0.2 in univariate models. The whole model (with all predictors) was statistically significant (*p* = 0.016). There was no significant multicollinearity between the predictors. In the multivariate logistic regression model, statistically significant predictor of total RetTSQ score reduction was presence of hyperlipidaemia (B = 1.044; *p* = 0.021). The models predictors are listed in Table 5. 

## 4. Discussion

Our results indicate that the mean NEI VFQ-25 composite score prior to and following PRP for the whole study sample, does have a statistically significant effect on the patients’ VR-QOL. A quantitative assessment of our results revealed, that PRP leads to deterioration in the scores for the items “General vision” and “Dependency”. Other aspects of visual function examined with NEI VFQ-25 did not prove to have a statistically significant impact on VR-QOL. Our results are inconsistent with the results of Tsilimbaris et al. They reported that treatment with PRP does not have a significant effect on NEI VFQ-25 scores and that in their group of patients the mean values of the total score and the subscale scores of the NEI VFQ-25 questionnaire remained unchanged after treatment [19]. When interpreting these results, it should be taken into account that our study reveals a statistically significant decline in the patients’ BCVA of the better-sighted eyes post-treatment, while Tsilimbaris et al. in their study group found a small, but not statistically significant loss in patients’ visual acuity [19]. Considering the visual acuity deterioration in our sample, the discrepancies found between these studies are not surprising. Additionally, when interpreting these results, the size of the study sample definitely should be taken into account as a small sample size in some studies leads to a lack of power of the study.

Regarding the total RetTSQ score, our study did not show any difference in treatment satisfaction between pre- and post- PRP groups. Literature review shows significantly lower RetTSQ scores in previously performed laser treatments for DR compared to their counterparts [2,17], regardless of type of performed laser. Sub-scale analysis prior to and following laser photocoagulation showed a statistically significant change in five of the 13 items of RetTSQ: overall satisfaction with current treatment, discomfort/pain, unpleasantness of treatment, patient influence on the treatment and received information about the treatment. According to Ramu et al. the patients’ perceived treatment satisfaction was based on their final VA status, while baseline VA and macular thickness were not found to affect RetTSQ scores [20]. Considering the statistically significant decline in the patients’ post treatment visual acuity and the decrease of their TS score in our sample, it is not surprising that we found a similar result, in regard to their overall satisfaction with the treatment.

Many patients find PRP a painful experience, and a substantial number of patients are therefore undertreated and at an increased risk of developing blindness [21]. Additionally, the pre-laser dilation of pupils can lead to blurred vision for several hours after treatment. Additionally, eye drops given after procedure can lead to visual symptoms such as burning of the eye and other eye discomfort. All of these can affect treatment satisfaction and compliance of patients undergoing this therapeutic procedure. Our study confirmed these facts in the RetTSQ questionnaire by having the scores of discomfort/pain and unpleasantness of treatment significantly reduced. 

Moreover, findings from our study suggest that diabetic patients received deficient information for their treatment process from health workers. This was shown by a statistically significant change in the RetTSQ score for the received information about treatment item before and after PRP treatment, i.e., patients showed a lower degree of satisfaction after the laser procedure for this item. Given that the treatment plan should be individualized and performed in cooperation with the patient and their family on one side and the physician on the other, our results indicate a broader problem of awareness among healthcare professionals of the importance of providing patients with information about their treatment. Likewise, our study demonstrated that patients with DR frequently felt like they were not involved in the treatment decision-making, since they had a lower satisfaction score in the item of patients’ influence on the treatment. Implementation of a management plan requires that each aspect is understood and approvedby the patient and physician, and that the goals and treatment plan are reasonable.

In our study, the severity of the treatment, as indicated by the mean number of laser applications given at each treatment session, was correlated with the change in composite NEI VFQ-25 post treatment score. The variable number of laser burns has an odds ratio of 0.997. This shows that subjects with each burn have a 0.3% lower risk of reducing the composite score, with control of all other factors in the model. Our results are inconsistent with the results of Tsilimbaris et al., who noted that in the range of treatments their patients received, there was no significant connection of the treatment intensity with the effect on VR-QOL [19]. This difference could be explained by their shorter follow-up period in which their patients did not have time to develop a decrease in their visual acuity following PRP. 

In our study sample, according to the RetTSQ questionnaire, the presence of hyperlipidaemia was a predictor of a worse outcome in TS after PRP relative to baseline values. The odds ratio (OR 2.840) for the variable hyperlipidaemia, with control of all other factors in the model, shows that subjects with hyperlipidaemia are two times more likely to reduce their total score. The presence of hyperlipidemia can lead to more pronounced leakage of retinal vessels and formation of hard exudate, which can be especially pronounced after PRP. According to Bryl et al. patients with the higher levels of hyperlipidemia are correlated with more advanced retinopathy and exudative diabetic macular oedema. [22]. Additionally, it is important to note that there is a significant positive correlation of observed serum levels of urea, creatinine and glycosylated haemoglobin with the severity of retinopathy [23]; indicating that when DR occurs in patients with DM, systemic conditions should be improved as much as possible while treating ocular conditions [24]. These results could be explained by the fact that patients who have an additional complication of hyperlipidaemia have more severe diabetic retinopathy, and therefore worse visual function. This results in them needing more extensive PRP treatment, which in turn leads to further short-term damage of their visual function. Finally, this can be summed up as the result of a longer duration of diabetes with higher levels of glycosylated haemoglobin.

There are strengths and limitations to our study. The strengths of this investigation are reflected in its prospective design and the systematic evaluation and review of the participants whose medical and functional characteristics were evaluated and recorded with the aid of valid instruments. The patients’ subjective perception of visual function was evaluated by means of NEI VFQ-25, a tool that has been tested and proven to be reliable and useful for group-level comparisons of VR-QOL. On the other hand, there is no comparable data from other studies that used the RetTSQ as we did, in patients with PDR treated with PRP. The fact that the RetTSQ has previously been employed in only a few studies [17,20] and has not been rigorously tested in more researches represents one of the main limitations of our study [25]. Further testing of RetTSQ is required to establish the usefulness of RetTSQ to measure patient satisfaction of different treatments. It is also important to emphasize the need for studies with a larger number of subjects that would include different ethnic groups.

## 5. Conclusions

The use of vision-related quality of life and treatment satisfaction questionnaire in conjunction with clinical examination appears to provide a more comprehensive overview of an individuals’ daily well-being following laser photocoagulation. The findings of the current study indicate that laser treatment for diabetic retinopathy can lead to a deterioration of some of the patients’ perceived functional status, quality of life and treatment satisfaction. Health-care providers should inform patients about their treatment options and together decide which therapeutic method is best for them. New studies are needed to compare patients reported outcomes with other treatment modalities, alone or in combination with PRP, to be able to determine the impact of vision loss on patients’ daily function and general well-being, which could improve their clinical management and accomplish better outcomes. 

## Figures and Tables

**Table 1:** Demographic and clinical characteristics of the study participants.

Variables	Sample, n = 95
Age (years), mean ± SD	57.4 ± 13.2
Gender, Male, number (%)	70 (73.7)
Marital status, number (%)	
Married	61 (65)
Other	34 (35)
Educational status, number (%)	
Elementary school (1–8 years)	17 (17.9)
Secondary school	63 (66.3)
Higher school	7 (7.4)
University degree	8 (8.4)
Working status, number (%)	
Working	34 (35.8)
Not working	16 (16.8)
Pensioner	45 (47.4)
Smoking status, number (%)	
Yes	18 (18.9)
No	53 (55.8)
Former smoker	24 (25.3)
Physical activity (more than 30 min per day), number (%)	
Yes	53 (55.8)
No	42 (44.2)
Visual acuity (Snellen), mean ± SD	
Better eye	0.62 ± 0.31
Worse eye	0.43 ± 0.36

Abbreviation: SD, standard deviation.

**Table 2:** Comparison of NEI VFQ-25 Mean Subscale and Composites scores prior to and following laser photocoagulation.

Scale	Before (Mean ± SD)	After (Mean ± SD)	*p*-Value
General health	36.1 ± 26.5	37.1 ± 25.8	0.635
General vision	54.3 ± 19.5	45.9 ± 25.3	<0.001
Ocular pain	84.0 ± 18.2	82.2 ± 17.8	0.258
Near activities	63.2 ± 24.7	63.3 ± 23.3	0.929
Distance activities	72.9 ± 23.5	72.9 ± 25.3	0.979
Social functioning	84.3 ± 21.4	83.7 ± 23.0	0.678
Mental health	53.1 ± 28.3	52.0 ± 28.0	0.531
Role difficulties	64.6 ± 31.1	61.7 ± 31.3	0.085
Dependency	77.8 ± 27.4	73.8 ± 30.0	0.037
Driving	76.5 ± 25.0	75.2 ± 20.2	0.290
Colour vision	88.0 ± 21.7	86.1 ± 21.4	0.239
Peripheral vision	74.2 ± 26.7	71.6 ± 27.0	0.198
Composite score	65.4 ± 17.4	63.3 ± 19.5	0.045

Abbreviation: SD, standard deviation.

**Table 3:** RetTSQ score change between baseline and the exit visit in all items, using paired samples test.

**RetTSQ Subscales**	Pretreatment Score	Posttreatment Score	Mean	SD	*p*-Value
Overall satisfaction with current treatment	5.02	4.54	−0.48	2.10	0.027
How well the treatment works	4.79	4.49	−0.30	1.74	0.103
Side effects;	4.45	4.57	0.12	2.19	0.608
Discomfort/pain	3.46	4.39	0.93	2.33	<0.001
Unpleasantness of treatment	3.58	4.29	0.71	2.47	0.006
Ease/difficulty of the treatment	4.06	4.50	0.44	2.28	0.062
Apprehension about the treatment	3.79	3.34	−0.45	2.64	0.099
Patient influence on the treatment	4.91	4.34	−0.57	2.28	0.015
Safety of the treatment	5.34	5.35	0.01	1.31	0.938
Time-taken	3.96	4.06	0.11	2.28	0.654
Received information about treatment	5.48	4.88	−0.60	1.64	0.001
Encouraging others with diabetic eye problems	5.81	5.78	−0.03	0.89	0.731
Continuity of treatment	5.64	5.49	−0.15	1.30	0.271

Abbreviations: RetTSQ, retinopathy treatment satisfaction questionnaire; SD, standard deviation.

**Table 4:** Multivariate logistic regression analysis for factors affecting NEI VFQ-25.

Factors Predicting Change in NEI VFQ-25	Multivariate Logistic Regression		
	B	OR (95% CI)	*p*-Values
Mean number of laser spots	−0.003	0.997 (0.996–0.999)	0.002
Nephropathy (Yes/No)	1.072	2.922 (0.906–9.425)	0.073
Smoking (Yes/No)	−0.336	0.714 (0.272–1.876)	0.495
Hyperlipidaemia (Yes/No)	0.384	1.468 (0.545–3.953)	0.447
Polineuropathy (Yes/No)	0.780	2.181 (0.788–6.039)	0.133

**Table 5:** Multivariate logistic regression analysis for factors affecting RetTSQ.

Factors Predicting Change in RetTSQ	Multivariate Logistic Regression		
	B	OR (95% CI)	*p*-Values
Physical activity (Yes/No)	−0.552	0.576 (0.241–1.378)	0.215
Hypertension (Yes/No)	−1.109	0.330 (0.091–1.190)	0.090
hyperlipidaemia (Yes/No)	1.044	2.840 (1.168–6.907)	0.021
Presence of DMO on better eye (Yes/No)	−0.655	0.519 (0.216–1.251)	0.144

Abbreviations: RetTSQ, retinopathy treatment satisfaction questionnaire; DMO, diabetic macular oedema.

## Data Availability

The datasets used and/or analysed during the current study are available without restriction from the corresponding author. All relevant data are within the paper.

## Appendix A

Patients from this study who already had DMO (in addition to PDR) and who met the criteria for pharmacological treatment with vascular endothelial growth factor (VEGF) inhibitors (recommended by the Serbian health authorities) were immediately referred to the Committee that made the decision on the use of VEGF inhibitors in the treatment of DMO. The procedure itself for obtaining a permit takes an average of two to three months, and in that period the laser treatment of these patients and their participation in the study was completed. After obtaining permission, pharmacological treatment of DME was started. It is the standard procedure of pharmacological treatment of DMO in Serbia.

## Appendix B

The version of RetTSQ that we used in this study includes one small but important change from the one used in the original development. The original “time consuming” item was reworded, as noted by Brose and Bradley 2009 [2], so that instead of asking about how time consuming the treatment was we ask how satisfied the respondent is with the time taken by the treatment. This enables respondents to express satisfaction with a treatment even if it is time consuming as well as being possible to express dissatisfaction. This ‘time taken’ wording is the same as that which is used in the MacTSQ for macular disease and there the item loads on the positive subscale that Karadzic et al. [17] found in their working with RetTSQ. This makes sense when we realize that the version of RetTSQ is not the original with a “time-consuming” item, but rather is the revision with the “time-taken” item. In this paper, we relabeled that item accordingly as ‘time taken’. Certainly, the overall scale is not affected and this is what we have reported in this paper, along with the individual items of the RetTSQ in our current manuscript so there are therefore no changes in this respect.
